# A Comparative Assessment of Mechanisms and Effectiveness of Radiosensitization by Titanium Peroxide and Gold Nanoparticles

**DOI:** 10.3390/nano10061125

**Published:** 2020-06-07

**Authors:** Mennaallah Hassan, Masao Nakayama, Mohammed Salah, Hiroaki Akasaka, Hikaru Kubota, Makiko Nakahana, Tatsuichiro Tagawa, Kenta Morita, Ai Nakaoka, Takeaki Ishihara, Daisuke Miyawaki, Kenji Yoshida, Yuya Nishimura, Chiaki Ogino, Ryohei Sasaki

**Affiliations:** 1 Division of Radiation Oncology, Kobe University Graduate School of Medicine, 7-5-2 Kusunokicho, Chuo-ku, Kobe 650-0017, Japan; menna_alesawy@ymail.com (M.H.); naka2008@med.kobe-u.ac.jp (M.N.); drsalahbio@yahoo.com (M.S.); akasaka@harbor.kobe-u.ac.jp (H.A.); hk.0113hu@gmail.com (H.K.); nakahana-m@people.kobe-u.ac.jp (M.N.); ttagawa@med.kobe-u.ac.jp (T.T.); nakaoka@med.kobe-u.ac.jp (A.N.); take3036@med.kobe-u.ac.jp (T.I.); miyawaki@med.kobe-u.ac.jp (D.M.); soundandvision@outlook.jp (K.Y.); 2 Department of Clinical Oncology, Faculty of Medicine, Sohag University, Sohag 82524, Egypt; 3 Discipline of Medical Radiations, School of Biomedical & Health Sciences, RMIT University, Bundoora Campus, Victoria 3083, Australia; 4 Department of Biochemistry, Faculty of Veterinary Medicine, South Valley University, Qena 83522, Egypt; 5 Department of Chemical Science and Engineering, Graduate School of Engineering, Kobe University, 1-1 Rokkodaicho, Nada-ku, Kobe 657-8501, Japan; moyashi.batake@gmail.com (K.M.); nyuya@landscape.kobe-u.ac.jp (Y.N.); ochiaki@port.kobe-u.ac.jp (C.O.); 6 Research Facility Center for Science and Technology, Kobe University, 1-1 Rokkodaicho, Nada-ku, Kobe 657-8501, Japan

**Keywords:** nanoparticles, non-cytotoxic, radiosensitization, gold nanoparticles, titanium peroxide nanoparticles, reactive oxygen species, oxidative stress

## Abstract

The development of potentially safe radiosensitizing agents is essential to enhance the treatment outcomes of radioresistant cancers. The titanium peroxide nanoparticle (TiOxNP) was originally produced using the titanium dioxide nanoparticle, and it showed excellent reactive oxygen species (ROS) generation in response to ionizing radiation. Surface coating the TiOxNPs with polyacrylic acid (PAA) showed low toxicity to the living body and excellent radiosensitizing effect on cancer cells. Herein, we evaluated the mechanism of radiosensitization by PAA-TiOxNPs in comparison with gold nanoparticles (AuNPs) which represent high-atomic-number nanoparticles that show a radiosensitizing effect through the emission of secondary electrons. The anticancer effects of both nanoparticles were compared by induction of apoptosis, colony-forming assay, and the inhibition of tumor growth. PAA-TiOxNPs showed a significantly more radiosensitizing effect than that of AuNPs. A comparison of the types and amounts of ROS generated showed that hydrogen peroxide generation by PAA-TiOxNPs was the major factor that contributed to the nanoparticle radiosensitization. Importantly, PAA-TiOxNPs were generally nontoxic to healthy mice and caused no histological abnormalities in the liver, kidney, lung, and heart tissues.

## 1. Introduction

The use of nanoparticles (NPs) in biology and medicine is rapidly increasing. Their unique properties and size enable their use in several biological applications, such as cancer diagnosis and treatment, drug and gene delivery, detection of pathogens, and tissue engineering [1]. Recently, considerable progress has been made in the use of NPs as tools for cancer therapy [2,3]. In the field of radiation oncology, some nanoparticles showed the ability to sensitize cancer cells to radiation damage [4]. Despite the major advances in radiation delivery techniques, the toxicity to the surrounding normal tissue is a major dose-limiting factor, especially for radioresistant tumors and/or tumors near vital organs. As the radiation damage occurs due to a direct damage of the cellular targets (about 30%) and indirect damage (about 70%) through the production of free radicals, it seems ideal to harness the NPs that show a photocatalytic activity and an ability to generate free radicals to enhance the radiation effect [5].

Inorganic NPs are considered to achieve higher tumor concentrations due to the enhanced permeation and retention effect [6]. Moreover, some NPs react with ionizing radiation (IR), and scatter and emit secondary electrons [4]. A variety of NPs have been investigated as candidates for radiosensitization, including gold (Au), silver, gadolinium, hafnium oxide (HfO_2_), and titanium dioxide (TiO_2_) [7,8,9,10,11]. Even though their cytotoxic effect on cancer cells is significantly favorable, it is necessary to ensure that they are nontoxic to healthy body organs and are potentially safe before clinical use. AuNPs have been investigated as radiosensitizers. In 2004, Hainfeld et al. initially showed the ability of AuNPs to enhance the radiation effect in mice with kilovoltage radiation [12]. This work was followed by further studies that showed the radiosensitizing ability of AuNPs [13]. Researchers assume this radiosensitizing effect to be due to the high atomic number and mass energy coefficient of AuNPs, leading to an enhancement in the photoelectric and Auger electron effects. These secondary electrons damage the cell targets directly or ionize water molecules to produce reactive oxygen species (ROS), mainly hydroxyl radicals (HO˙) [14,15,16]. Moreover, the effects of AuNPs on cell cycle phases, induction of deoxyribonucleic acid (DNA) damage, and inhibition of DNA repair mechanisms are additional mechanisms for AuNP radiosensitization [16]. Although AuNPs can enhance radiation damage with low energy radiation, their radiosensitizing effect with megavoltage radiation remains controversial [17,18]. Reports on Au nanotoxicity are contradictory. The toxicity of the AuNPs depends on their size, shape, and surface chemistry (including the hydrophobicity and charge) [19,20,21]. Importantly, nephrotoxicity and hepatotoxicity of AuNPs are of major concern. Small AuNPs (5–10 nm) have been reported to damage liver, lung, and renal tissues after in vivo administration in rats [22,23]. In the same setting, administration of various natural antioxidants has been shown to protect against AuNP-mediated nephrotoxicity, hepatotoxicity, lipid peroxidation, oxidative stress, and inflammatory damage to the liver and kidney tissues suggesting that Au-nanotoxicity is mediated through ROS generation and oxidative stress [24,25]. Thus, identification of less- or nontoxic agents is essential.

Titanium peroxide nanoparticle (TiOxNPs) are synthesized from the anatase-type TiO_2_NPs after hydrogen peroxide (H_2_O_2_) treatment and surface coating with polyacrylic acid (PAA) [26]. They show the ability to continuously generate or release H_2_O_2_ into the liquid phase of a dispersion. Coating with PAA prevents NP aggregation and allows a homogenous dispersion in aqueous medium. This provides a stable dispersion at neutral pH and high salt concentration [27]. Despite the low atomic number of PAA-TiOxNPs in comparison with that of AuNPs, they showed the ability to significantly enhance the radiation effects [28]. First, their radiosensitizing effect was reported to the production of highly reactive ROS (HO˙ radicals), and the contribution of H_2_O_2_ generation in enhancing radiation-induced damage required further investigation. In the following study, H_2_O_2_ generation was enhanced by a combination of PAA-TiOxNPs and irradiation. Previously, this combination showed potential safety after intravenous injection in either healthy or xenografted mice, as observed by the nonsignificant increase in the biochemical blood tests of the liver and kidney functions [29].

In this study, we first investigated whether the TiOxNPs might be toxic or not on the normal organs of healthy mice through histological examinations of the liver, kidney, lung, and heart tissues. Subsequently, the radiosensitizing effect of TiOxNPs was evaluated in comparison with that of AuNPs, with regard to the types of ROS generated and the cytotoxic effects induced in vitro and in vivo. Moreover, we evaluated the biological responses occurring within the tumor xenografts to compensate for the increased oxidative stress. Understanding the different mechanisms of radiosensitization by different NPs enables us to choose the best radiosensitizing agent for each cancer type and tailor the treatment according to the tumor’s biological behavior.

## 2. Materials and Methods

### 2.1. Preparation of Nanoparticles

TiOxNPs were synthesized from anatase-type TiO_2_NPs according to previously reported procedures that involve H_2_O_2_ processing [26,27]. For in vitro and in vivo experiments, the surfaces were modified using PAA. The material for the TiO_2_NPs (STS-01) was purchased from Ishihara Sangyo, Ltd. (Osaka, Japan). The AuNPs were purchased from BBI solutions (EMGC50, BBI Solutions, batch number 023844-F3, Cardiff, UK). Table 1 describes the physicochemical similarities and differences between AuNPs and PAA-TiOxNPs.

### 2.2. Cell Culture and Animal Care

The MIA PaCa-2 JCRB0070 human pancreatic cancer cell line was obtained from the American Type Culture Collection (Rockville, MD, USA). Original stocks were verified by the National Institute of Biomedical Innovation-Japanese Collection of Research Bioresources Cell Bank to be mycoplasma-, bacteria-, and fungi-free and were further authenticated by short tandem repeat mapping. MIA PaCa-2 cells were maintained in Minimum Essential Medium Eagle (Sigma Aldrich, Irvine, United Kingdom) supplemented with 10% fetal bovine serum and 1% penicillin-streptomycin.

C57/BL6 mice (eight weeks old) and male immunodeficient BALB/cAJcl nude mice (body weight: 20–22 g and six weeks old) were purchased from CLEA corporation (CLEA, Inc., Tokyo, Japan). The nude mice were maintained in specific pathogen-free animal care facilities. Housing under 21–25 °C and 40–70% humidity with free access to food and water was maintained. All animal experiments were approved by the Kobe University Institutional Animal Care and Use Committee (permission number: P130614) and performed in accordance with the Kobe University Animal Experimentation Regulations.

### 2.3. Experimental Design

For the toxicity experiment, C57/BL6 mice were divided randomly into two groups, the control group and TiOxNPs group. Three mice were used in each group. Control group treated with phosphate-buffered saline (PBS), while TiOxNPs group treated with PAA-TiOxNPs as demonstrated below. The mice were inspected for any abnormalities at days 1, 2, 3, 7, 14, and weekly thereafter. After 60 days, the mice were euthanized for normal organ evaluation.

For the xenograft assay, MIA PaCa-2 xenografted nude mice were randomly divided into six treatment groups: the control group, AuNPs alone, TiOxNPs alone, 5 Gy X-ray irradiation alone, AuNPs combined with 5 Gy irradiation, and TiOxNPs combined with 5 Gy irradiation (as described below). Each group consisted of four mice. The nanoparticles were administered intratumorally followed by 5 Gy x-irradiation of the tumors after one hour then, body weight and tumor volume were followed for 55 days. Moreover, on day 1, the induction of apoptosis and the antioxidant enzymes expression level were evaluated in tumor tissues. On days 7 and 55, tumor tissues were collected to evaluate the antioxidant enzymes expression level. The ability to enhance ROS generation under x-irradiation was evaluated in a cell-free system, in MIA PaCa-2 cells and in vivo.

### 2.4. Normal Organ Toxicity

PAA-TiOxNPs were resuspended in phosphate-buffered saline (PBS) solution at concentration 18 mg/mL and three C57/BL6 mice were injected intravenously with the NP suspension via the tail vein at a dose of 90 mg/kg body weight. Three control mice were injected with 100 µL of PBS. All mice were observed for 60 days for any abnormalities. On day 60, the mice were anesthetized with isoflurane and euthanized. The liver, kidneys, lungs, and heart were collected. Tissues were fixed with 10% formalin and embedded in paraffin blocks. Sections were prepared for hematoxylin-eosin (H&E) staining. Bright field images were acquired using a BZ-9000 microscope (Keyence, Japan) to detect any microscopic abnormalities compared with the control mice.

### 2.5. X-ray Irradiation

X-ray irradiation was performed using the MBR-1505R2 X-ray generator (Hitachi, Tokyo, Japan) at a voltage of 150 kV and a current of 5 mA with a 1-mm thick aluminum filter (0.5 Gy min^−1^ at the target). Prior to each experiment, the mice were anesthetized using an intraperitoneal administration of somnopentyl (0.1 mg g^−1^ body weight), and then immobilized in a customized harness that exposed the tumor while shielding the remainder of the body with lead during irradiation, as described previously [31].

### 2.6. ROS Evaluation

In a cell-free system, measurements of ROS generation in response to X-ray irradiation were performed using three different chemical probes. Concentrations of PAA-TiOxNPs in this experiment were 50, 100, 200, and 400 µg mL^−1^, and those of AuNPs were 1.5, 2, 4, and 15 µg mL^−1^. The amount of HO˙ radicals was measured by 3′-(p-aminophenyl) fluorescein (APF) (Sekisui Medical Co. Ltd., Tokyo, Japan) [32]. To detect superoxide anions (O2˙), 50 ng mL^−1^ of dihydroethidium (dHE) (Molecular Probes, Inc., OR, USA) was used. The NP suspensions were then exposed to 0, 5, and 10 Gy of IR. The APF and dHE signals were measured using a multiwell plate reader (EnSpire label-free microplates, PerkinElmer, MA, USA) at excitation/emission wavelengths of 485/538 nm for APF and 485/590 nm for dHE. The possibility of quenching of the fluorescent agent by the NPs should be considered during the interpretation of ROS generation by a fluorescence assay. Therefore, we measured the amount of H_2_O_2_ generation using the absorbance intensity of BIOXYTECH H_2_O_2_-560 reagent according to the manufacturer’s protocol (OXIS International, Portland, OR, USA). The absorbance was measured using a spectrophotometer (EnSpire label-free microplates, PerkinElmer, MA, USA) at 560 nm.

ROS generation was evaluated in MIA PaCa-2 cells as follows: the cells seeded at a density of 2.5 × 10^5^ cells/well in six-well plates and treated with PAA-TiOxNPs at a final concentration of 200 µg mL^−1^ and AuNPs at a final concentration of 15 µg mL^−1^, followed by incubation for 1 h at 37 °C with/without 5 Gy of irradiation. The cells were stained with 50 μM carboxy-2′,7′-dichlorofluorescein (C-H_2_DCF) (Molecular Probes, Inc., Eugene, OR, USA) per sample, incubated for 45 min, and then stained with Hoechst (1:2000, Invitrogen, Eugene, OR, USA) for nuclear staining. The degree of fluorescence of C-H_2_DCF was detected using the BZ-9000 fluorescence microscope (Keyence, Japan). Different fields were imaged for each sample and three samples were used for each treatment group. C-H_2_DCF-positive cells were counted using the ImageJ software (version 1.8.0).

### 2.7. Colony-formation Assay

The colony-formation assay was performed to assess in vitro cytotoxicity as demonstrated previously [33]. Briefly, MIA PaCa-2 cells (2.5 × 10^5^) were treated with PAA-TiOxNPs at final concentrations of 150, 200, and 400 µg mL^−1^ or AuNPs at final concentrations of 1.5, 2, 4, and 15 µg mL^−1^, incubated for 1 h, and then irradiated with 0, 2, or 5 Gy of irradiation. The treated cells were counted and replated onto a new six-well plate with a fresh medium without NPs and seeded at a density of 200–8000 cells/well. The cells were incubated for two weeks until the cell population completed colony formation. Fixation with acetic acid/methanol (1:7) and staining with 0.5% crystal violet solution (Sigma Aldrich) were performed. Colonies consisting of more than 50 cells were counted, and the surviving fractions were calculated based on the survival of nonirradiated cells.

### 2.8. Xenograft Assay

MIA PaCa-2 cells (2 × 10^6^ cells) were injected subcutaneously into the hind legs of the BALB/cAJcl nude mice. Once the tumor volume reached 100–200 mm³ using the formula L × W²/2, where L is the longest axis, and W is the shortest axis of the tumor, naïve mice were randomly assigned into six groups: the control group (treated with phosphate-buffered saline), AuNPs alone (treated with AuNPs suspension at a concentration of 15 µg mL^−1^), TiOxNPs alone (treated with PAA-TiOxNPs suspension at a concentration of 1500 µg mL^−1^ [15 mg/kg body weight]), 5 Gy alone (treated with 5 Gy X-ray irradiation), AuNPs combined with 5 Gy (treated with AuNPs suspension at a concentration of 15 µg mL^−1^ and 5 Gy X-ray irradiation), and TiOxNPs combined with 5 Gy (treated with PAA-TiOxNPs suspension at a concentration of 1500 µg mL^−1^ [15 mg/kg body weight] and 5 Gy X-ray irradiation). The NPs were resuspended in PBS solution. No animal was excluded from the study. Tumor size, body weight, and health condition of all mice were followed every two to three days for 55 days post-treatment. On day 55, all mice were sacrificed for tumor tissue collection.

### 2.9. TUNEL Assay In Vivo

Tumor tissues were excised at 24 h post treatment, fixed in 10% formalin, and embedded in paraffin sections (4 μm thick). Induction of apoptosis was evaluated using the terminal deoxynucleotidyl transferase dUTP nick end labeling (TUNEL) assay according to the manufacturer’s protocols (Roche Applied Science, Indianapolis, IN, USA). The nuclei were stained with 4′,6-diamidino-2-phenylindole (DAPI). TUNEL-positive signals were detected using the BZ-9000 fluorescence microscope (Keyence, Japan) as described previously [34], and counted using ImageJ software (version 1.8.0, Bethesda, MD, USA) for each treatment. Each treatment group was represented by three slides and the number of apoptotic cells was calculated by averaging the number of TUNEL-positive signals from different fields per each slide.

### 2.10. Immunohistochemical (IHC) Analysis

Sections of paraffin-embedded tumor tissues of the six treatment groups at days 1, 7, and 55 after treatment were deparaffinized and stained using the peroxidase-anti-peroxidase (PAP) IHC method (Dako REAL peroxidase blocking solution S2023, Glostrup, Denmark) with an anticatalase antibody (1:50, Abcam, ab16731) and antiglutathione peroxidase antibody (1:100, Abcam, ab22604). Nuclei were counterstained using the hematoxylin stain (Mayer’s hematoxylin solution, Muto Pure Chemicals Co., Tokyo, Japan).

### 2.11. Statistical Analysis

Data are expressed as the mean ± standard deviation. Comparisons of the NP suspensions in cell-free experiments and the treatment groups for H_2_O_2_ production in vitro were performed using two-way ANOVA with Tukey’s multiple comparison test. However, Student’s t-test was used in the colony-formation assay to determine the difference between the MIA paCa-2 cells treated with X-ray irradiation alone and those treated with NPs and X-ray irradiation. For in vivo experiments, two-way ANOVA with Tukey’s multiple comparison test was also used to compare the different groups of mice for tumor growth inhibition. Differences were considered significant at the 95% confidence interval (*p* < 0.05).

## 3. Results

### 3.1. Assessment of Normal Organs after Intravenous Injection

**Figure 1:**
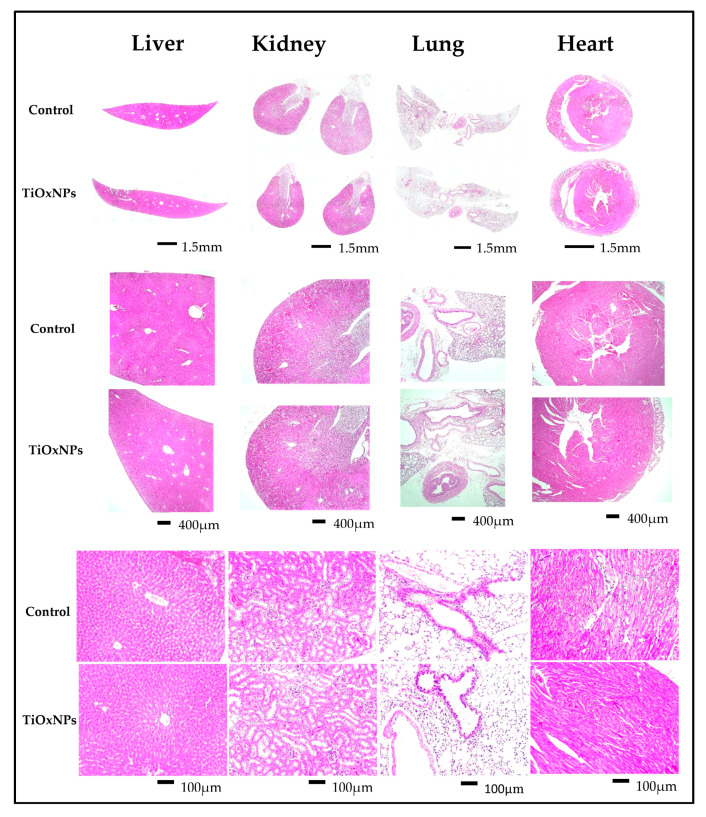
Assessment of organ toxicity after intravenous injection of polyacrylic acid titanium peroxide nanoparticles (PAA-TiOxNPs) in healthy C57/BL6 mice. No apparent microscopic abnormalities were observed in the PAA-TiOxNP-treated mice compared with the control mice. Scale bars: upper panel, 1.5 mm; middle panel, 400 µm; lower panel, 100 µm.

To assess the safety of TiOxNPs after intravenous injection in healthy mice, the liver, kidneys, lungs, and heart were evaluated grossly and microscopically after H&E staining and compared with those of the control group. The gross appearance (shape, color, surface, and weight) of liver, kidneys, lungs, and heart were quite similar between control mice and PAA-TiOxNPs-treated mice. Figure 1 shows no aberrant gross or microscopic observations in the tissues of PAA-TiOxNPs-treated mice and the control mice. The liver tissue shows normal liver architecture with preserved anatomy of hepatic lobules. There are no signs of inflammation or fibrosis. The kidneys show no pathological abnormalities, the renal glomeruli are preserved, and the renal tubules look normal. The lung tissue shows patent alveolar sacs with intact epithelium and no signs of inflammation or fibrosis. The heart tissue shows no signs of congestion, inflammation, or fibrosis. In addition, none of the six mice died during the 60-day observation period which is in correlation with the follow up period of tumor growth inhibition in xenograft assay as described before.

### 3.2. ROS Production under X-ray Irradiation in Cell-Free System

**Figure 2:**
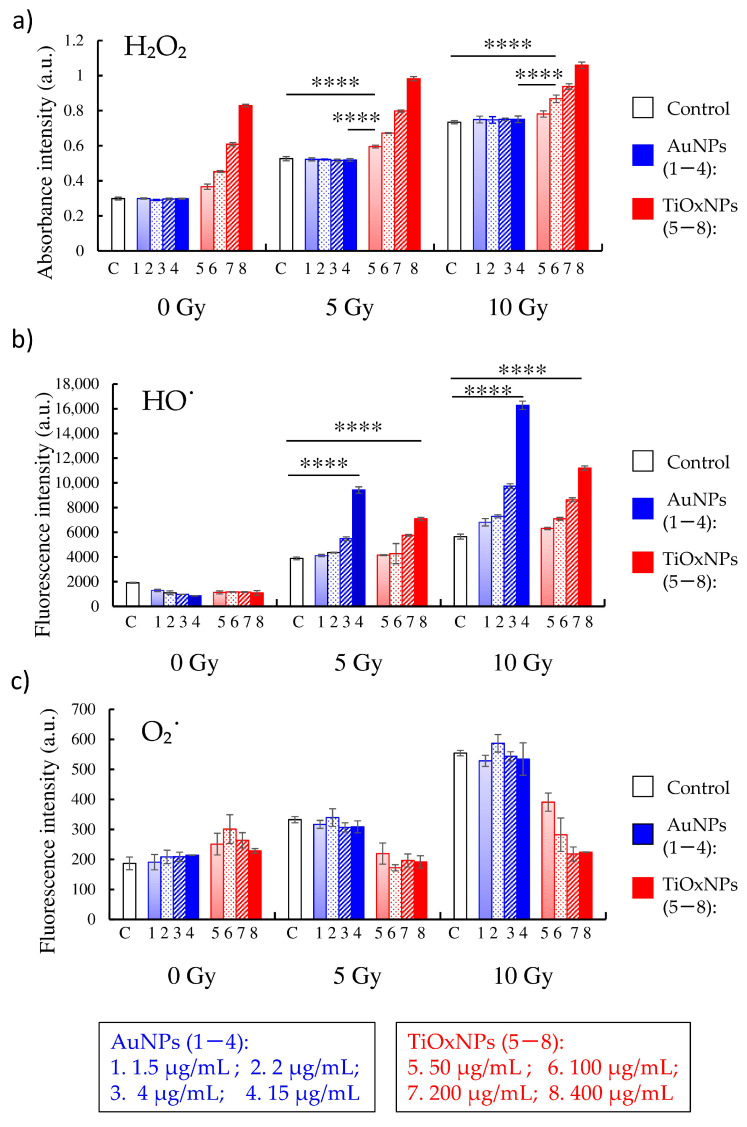
Reactive oxygen species (ROS) generation by different concentrations of titanium peroxide (TiOxNPs) and gold (AuNPs) nanoparticles in the cell-free system. (**a**) The absorbance intensity of the H_2_O_2_-560 reagent indicating the highest production of hydrogen peroxide (H_2_O_2_) by TiOxNPs compared with AuNPs or the control. (**b**) Fluorescence intensity of, 3′-(p-aminophenyl) fluorescein (APF) indicating that AuNPs enhanced hydroxyl (HO˙) radical production more than TiOxNPs. (**c**) Dihydroethidium (dHE) fluorescence intensity showing O_2_˙ production was not increased by TiOxNPs or AuNPs. Radiation doses were 0, 5, and 10 Gy. Data are shown as the mean ± standard deviation from five independent experiments. **** *p* < 0.0001.

In cell-free experiments, PAA-TiOxNPs, in combination with x-irradiation, significantly enhanced both H_2_O_2_ and HO˙ generation in NP concentration-dependent and radiation dose-dependent trends, while AuNPs increased only HO˙ radicals (Figure 2a,b). The estimated concentration of H_2_O_2_ (µM) is shown in supplementary material (Figure S1). Production of O_2_˙ by irradiation in the PAA-TiOxNPs dispersing water was decreased in a concentration-dependent manner (Figure 2c). The possible explanation of this phenomenon has not been raised in this study and further investigations might be necessary to clarify the whole ROS reactions.

### 3.3. Intracellular ROS Production

**Figure 3:**
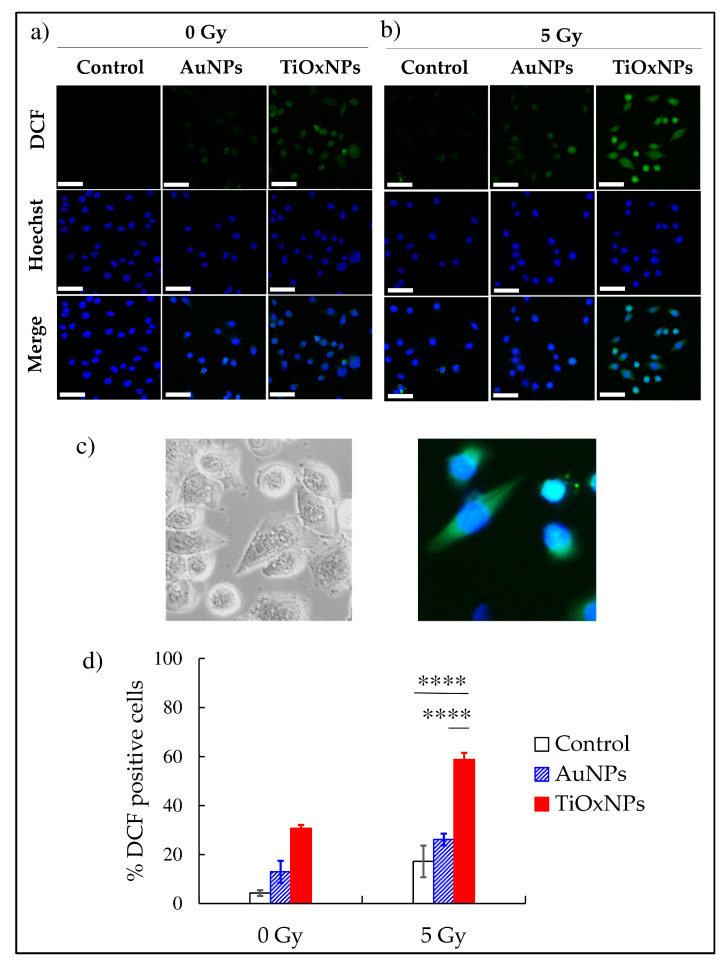
Assessment of hydrogen peroxide (H_2_O_2)_ production by titanium peroxide nanoparticles (TiOxNPs) and gold nanoparticles (AuNPs) under X-ray irradiation in MIA PaCa-2 cells. Fluorescence of H_2_O_2_ using a fluorescence microscope and, carboxy-2′, 7′-dichlorofluorescein (C-H_2_DCF) as fluorescent agent at (**a**) 0 Gy and (**b**) 5 Gy. (**c**) Phase contrast image of MIA PaCa-2 cells (on the left) and a magnified image for DCF-positive MIA PaCa-2 cells (on the right). (**d**) Graph representing the fluorescence of C-H_2_DCF in terms of mean and standard deviation. Scale bar = 50 µM, **** *p* < 0.0001.

We tested the ability of the NPs to induce H_2_O_2_ production in MIA PaCa-2 cells. In mice or humans, cells and organs develop several antioxidant defense systems to neutralize endogenous ROS. Cancer cells usually upregulate the antioxidant defense systems more than normal cells. Therefore, measuring the amounts of H_2_O_2_ in cancer cells after the radiation treatment is necessary to evaluate the potential of NPs as radiosensitizers. Interestingly, with 5 Gy of irradiation, PAA-TiOxNPs induced a significant increase in H_2_O_2_ production compared with either 5 Gy alone or AuNPs (both, *p* < 0.0001) (Figure 3a,b,d). The number of analyzed cells is supplemented in Table S1.

### 3.4. Enhancement of Radiation-Induced Cytotoxic Effect In Vitro

**Figure 4:**
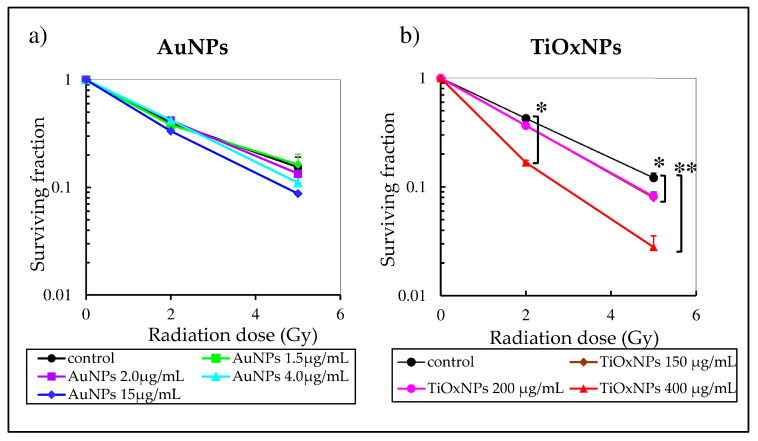
Assessment of cytotoxicity of titanium peroxide nanoparticles (TiOxNPs) compared with gold nanoparticles (AuNPs) combined with X-ray irradiation in vitro. (**a**) Colony-formation assay showing that combined treatment with AuNPs and 5 Gy radiation inhibited colony formation; however, this inhibition was not significant 0.05 < *p* < 0.1 (n = 3). (**b**) Colony-formation assay showing that combined treatment with TiOxNPs and 5 Gy radiation significantly inhibited colony formation, * *p* < 0.05, ** *p* < 0.01 (n = 4).

To compare cytotoxic effects of PAA-TiOxNPs with that of AuNPs, a colony-formation assay was performed. Treatment of MIA PaCa-2 cells with PAA-TiOxNPs, at concentrations of 150 and 200 µg mL^−1^, combined with 5 Gy significantly inhibited cell growth compared with radiation alone (*p* < 0.05 for both concentrations). In addition, treatment of MIA PaCa-2 cells with PAA-TiOxNPs, at concentrations of 400 µg mL^−1^, combined with either 2 or 5 Gy significantly inhibited colony formation compared with radiation alone (*p* < 0.05, *p* < 0.01 for 2 and 5 Gy respectively) (Figure 4b). In contrast, AuNPs, at concentrations 2, 4, and 15 µg mL^−1^ with 5 Gy showed a decrease in colony formation more than 5 Gy alone. However, this effect was not significant at any concentration (Figure 4a).

### 3.5. Tumor Growth Inhibitory Effect In Vivo

**Figure 5:**
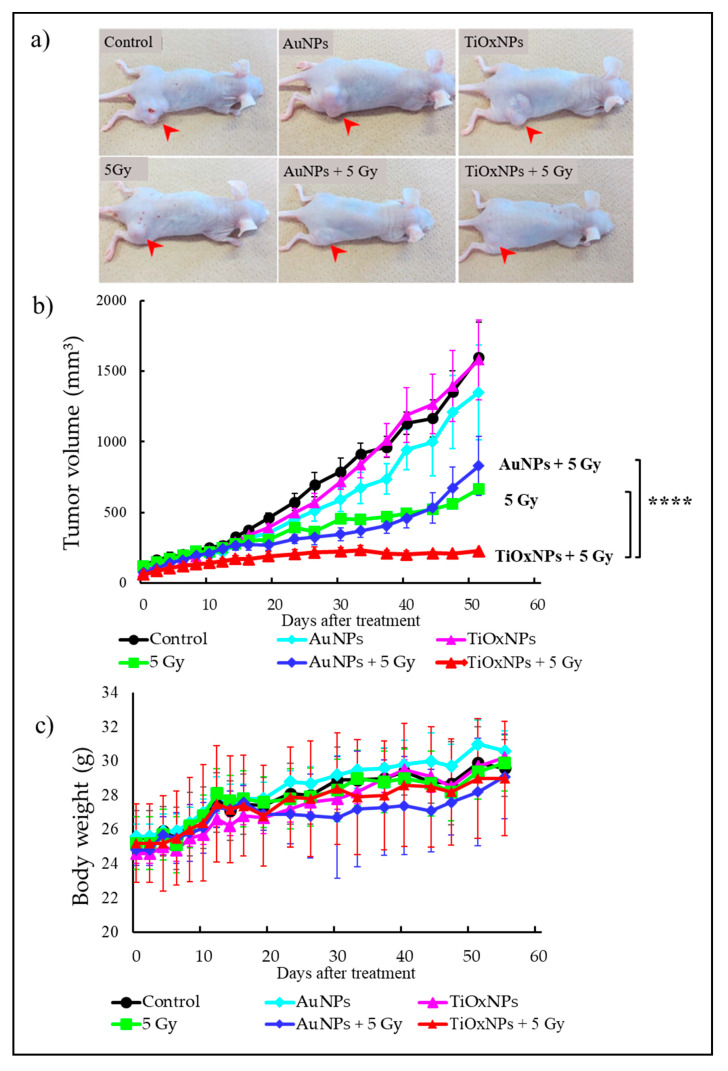
Assessment of tumor growth inhibitory effect of nanoparticles in vivo. (**a**) Tumor appearance in the xenografts for each treatment after 55 days (arrowhead). (**b**) Changes in tumor size after each treatment. (**c**) Changes in bodyweight of mice after each treatment. Data are shown as the mean ± 0.5 standard deviation. Data are shown as the mean ± 0.5 standard deviation. **** *p* < 0.0001 (n = 4).

MIA PaCa-2 xenografts were used for testing the effects of the NPs in vivo. TiOxNPs in combination with 5 Gy showed significantly greater radiation effects leading to higher tumor growth inhibition compared with that of 5 Gy alone or AuNPs with 5 Gy (both, *p* < 0.0001) (Figure 5a,b). These treatments were all well tolerated, as evidenced by no apparent loss of body weight (Figure 5c), and no mice died during the 55-day observation period.

### 3.6. Induction of Apoptosis In Vivo

**Figure 6:**
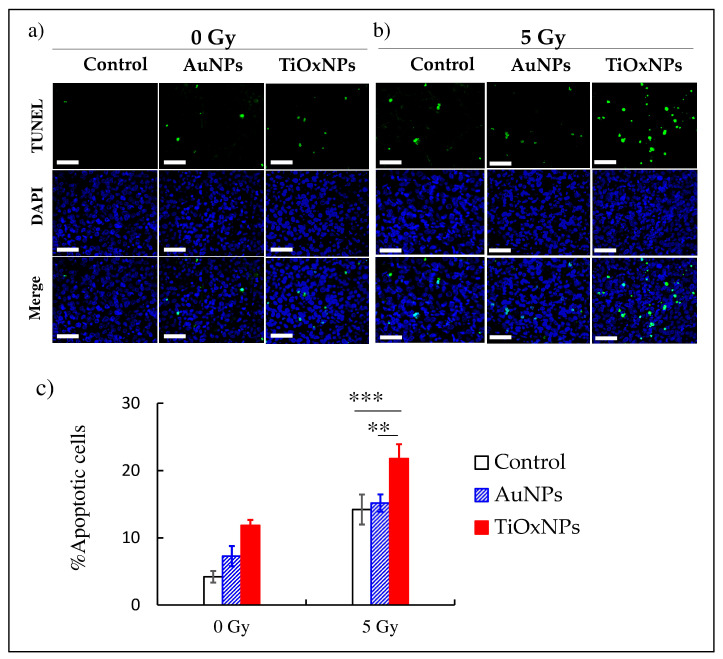
Detection of apoptosis induced by titanium peroxide (TiOxNPs) and gold (AuNPs) nanoparticles in vivo and its evaluation by terminal deoxynucleotidyl transferase dUTP nick end labeling (TUNEL) assay at (**a**) 0 and (**b**) 5 Gy. (**c**) Graph showing that the number of apoptotic cells with combined treatment of TiOxNPs and X-ray irradiation was significantly higher than that of AuNPs. Data are shown as the mean ± standard deviation. Scale bar = 50 µM, ** *p* < 0.01, *** *p* < 0.001.

Induction of apoptosis by NPs was evaluated in vivo. Without irradiation, both AuNPs and PAA-TiOxNPs administrations resulted in a slight increase in the number of apoptotic (TUNEL-positive) cells (Figure 6a). With 5 Gy of irradiation, consistent with the intracellular increase in H_2_O_2_ production, PAA-TiOxNPs induced a significant increase in the proportion of apoptotic cells compared with those by 5 Gy alone or AuNPs with 5 Gy (*p* < 0.001 and *p* < 0.01 respectively). The AuNPs had no significant increase of apoptosis (Figure 6b,c). The raw data of this experiment is supplemented in Table S2.

### 3.7. Antioxidant Enzymes: Catalase (Cat) and Glutathione Peroxidase (GPx-1) Expressions

**Figure 7:**
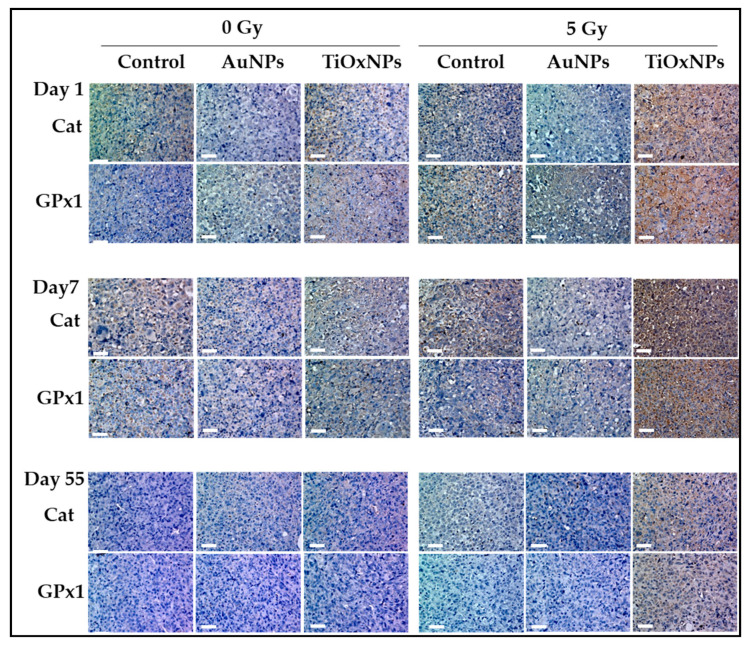
Immunohistochemical analyses of the antioxidant enzymes in the tumor tissue. The expression of catalase (Cat) and glutathione peroxidase (GPx1) enzymes using anticatalase and antiglutathione antibodies on day 1, day 7, and day 55. Scale bar = 50 µM.

To evaluate the cellular response to the increased oxidative stress, the expression of intracellular antioxidant enzymes, Cat and GPx1, were investigated by immunohistochemical analyses. It is noteworthy that both Cat and GPx1 expression were upregulated on days 1 and 7 in the group treated with TiOxNPs and 5 Gy, while no apparent increase was observed in other groups (Figure 7). The expression of antioxidant enzymes on day 55 was almost absent.

## 4. Discussion

Radiosensitization is one of the most promising applications for inorganic NPs and some NPs have been investigated as radiosensitizers. Radiotherapy plays an important role in cancer therapy, but there are several types of cancer or sarcoma that behave as radioresistant tumors. Treatment of these tumors is challenging and requires strategies to increase the radiation effect within the tumor, such as radiosensitization. A radiosensitizer is supposed to deliver higher radiation effects to the tumor without increasing damage dealt to the surrounding healthy tissues. The ultimate goal of combining NPs with radiation therapy is to increase the differential effect between healthy and tumor tissues. There are several inorganic NPs with different mechanisms of radiosensitization reported previously. However, the most intensively studied NPs are Au-based NPs. Although mechanisms of AuNPs for radiosensitization have been widely reported [16], the effectiveness against radioresistant tumors remains undetermined. TiOxNPs are newly developed NPs with unique properties. TiOxNPs have abilities to produce H_2_O_2_ molecules, in addition to HO˙ radicals, by X-ray radiation, as shown in Figure 2. To our knowledge, there are no direct comparisons between AuNPs and TiOxNPs investigating the mechanisms and efficacy of radiosensitization.

H_2_O_2_ is a ROS causing cell damage and apoptosis. It is converted to the HO˙ radical through the Fenton reaction [35]. HO˙ is considered to be an aggressive ROS causing DNA damage [36]. However, in the absence of the Fenton reaction, H_2_O_2_ accumulates within the lysosomes causing lysosomal membrane disruption, cell damage, and apoptosis [37]. As shown in Figure 3, the number of DCF-positive MIA PaCa-2 cells were significantly increased by PAA-TiOxNPs and irradiation indicating enhanced production of greater amounts of H_2_O_2_ in the MIA PaCa-2 cells. Consistently, H_2_O_2_ induction led to cytotoxic effects in vitro (Figure 4b), and induction of apoptosis in vivo (Figure 6), and eventually tumor growth inhibition in xenografts (Figure 5b). Our results, together, illustrated H_2_O_2_ acts as a mediator of radiosensitization by PAA-TiOxNPs. Notably, Ogawa and his colleagues have developed a new radiosensitizing method, named KORTUC, through directly injecting H_2_O_2,_ which showed excellent efficacy with superficially exposed and locally advanced radioresistant tumors and this strategy was tolerable for the treated patients [37,38,39]. The clinical outcomes brought by KORTUC support that the direct injection of H_2_O_2_ into a tumor in combination with radiotherapy could be a promising strategy to the radioresistant tumors. However, the KORTUC method consists of two injections per week and seems a time-consuming procedure for radiation oncologists. Therefore, if the PAA-TiOxNPs, as a radiosensitizer, act as continuous sources of H_2_O_2_, this strategy might be widely employed in the clinical settings.

AuNPs are commonly studied agents showing potent radiosensitizing abilities. Researchers have proposed that the ability of AuNPs to enhance radiation effects is due to their high atomic number. In the case of low-energy radiation, AuNPs interact with the incident photon and result in the emission of secondary electrons and photons [40,41,42]. These electrons, in turn, damage the biological molecules, directly or indirectly, through HO˙ free radical formation [41,42]. In line with previous reports, our results showed an enhanced HO˙ production by AuNPs in a cell-free system (Figure 2b). Although, Au is generally an inert material and considered nontoxic, the toxicity of AuNPs is still debated. According to Jia et al., this issue is due to the variations in particle sizes, shapes, and different surface modifications, in addition to variations in the cell lines and animal models examined, routes and doses of NP administration etc. [43]. They demonstrated AuNPs are toxic when used in biological systems in certain range of concentrations, however, proper surface modification may influence the toxic effects. Supplementary Table S3 summarizes some of the published studies involving factors promoting non-cytotoxicity of the AuNPs in vitro and in vivo [44,45,46,47,48,49,50,51,52]. It is noteworthy that there is a discrepancy between the tendency of HO˙ production by AuNPs in the cell-free system (Figure 2b) and their cytotoxicity showed by in vitro and in vivo experiments (Figure 4a and Figure 5b). The aforementioned tendency might be explained by the fates of ROS within the biological systems.

**Figure 8:**
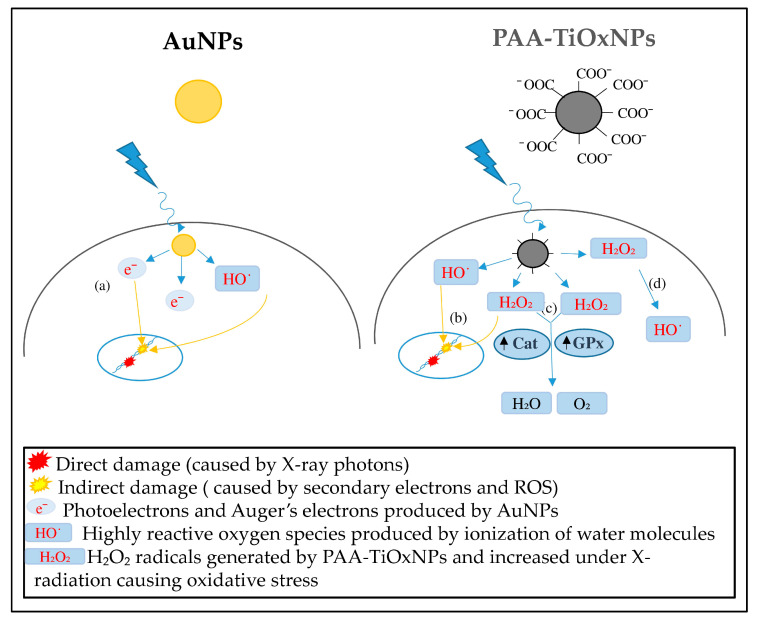
Scheme showing the X-ray photons causing direct (red stars) and indirect damage (yellow stars). (**a**) The radio-enhancing effect caused by AuNPs is due to the production of secondary electrons and HO˙ radicals that cause indirect damage to DNA and other cell components. (**b**) The radio-enhancing effect caused by PAA-TiOxNPs is due to the continuous production of large amounts of H_2_O_2_ increased by X-ray irradiation, which stimulates the (**c**) antioxidant defense system (Cat and GPx); however, the remaining H_2_O_2_ is converted to HO˙ radicals (**d**).

PAA-TiOxNPs sensitized MIA PaCa-2 cells to radiation damage more than AuNPs due to the increased apoptosis of PAA-TiOxNPs-treated cells (Figure 6). It has been reported that H_2_O_2_ can sensitize radioresistant cancer cells, such as those of osteosarcoma [53], prostate cancer [54], and melanoma [55], through radiation-induced damage and apoptosis. Production of ROS might be similar under the cell-free system, in cancer cells (in vitro), and in tumors (in vivo). However, the fates of ROS and their biological effects are believed to be different because cells possess various antioxidant defense systems to diminish ROS, and tumors may induce the iron-catalyzed Haber–Weiss reaction [56]. Thus, elevated ROS levels are usually counteracted by elevated antioxidant defenses to maintain redox homeostasis [57]. Redox homeostasis depends on a balance between the levels of oxidants and antioxidants [58]. Moreover, cancer cells can adapt to survive under certain levels of oxidative stress, which is called redox adaptation [59]. In our study, redox adaptation by Cat and GPx occurred in tumors treated with PAA-TiOxNPs and IR (Figure 7). Cat and GPx are the main antioxidant enzymes for H_2_O_2_. Increased expression of Cat and GPx reflects the continuous production of H_2_O_2_ by the treating agent in vivo. Notably, Cat and GPx expressions in PAA-TiOxNPs-treated tissues were observed on day 1. They increased to a maximum on day 7 after treatment, and then decreased on day 55. Meanwhile, the expressions of Cat and GPx were not observed in AuNP-treated tissues (Figure 7), indicating the inability to enhance H_2_O_2_ production. As a result, PAA-TiOxNPs and x-irradiation showed stronger tumor growth inhibitory effects than AuNPs and x-irradiation (Figure 5b). Induction of apoptosis, and increased expression of antioxidant enzymes, together, support that PAA-TiOxNPs are more powerful radiosensitizer through H_2_O_2_ production in vivo. Figure 8 is an illustrating scheme that shows the mechanisms of radiosensitization by AuNPs and PAA-TiOxNPs and the response of antioxidant enzymes (Cat and GPx).

Previously, it was reported that PAA-TiOxNPs showed potential accumulation within the liver after one week of injection, but this was associated with a nonsignificant increase in liver or kidney function blood tests as compared with that in the control [29]. Despite the low or no toxicity indicated by these standard tests, attention should be paid to the probability of cell/tissue damage. Therefore, potential damage to the liver, kidney, lung, and heart tissues after systemic administration of PAA-TiOxNPs was investigated in the current study at the histological level. Consistently, PAA-TiOxNPs showed no apparent cellular or tissue damage of these organs as compared with the control group (Figure 1). Our data and previous data [29], together, support PAA-TiOxNPs to be evaluated as nontoxic NPs in the later experiments within this study.

Coulter et al. [40] highlighted the gap between the wealth of preclinical data supporting high-atomic-number NPs as effective radiosensitizers, in addition to the few clinical studies and the lack of rigorous and systematic methodologies to evaluate NP efficacy. Recently, a promising radiosensitizing NP candidate, NBTXR3, was evaluated in a clinical multicenter, phase 2–3, randomized, controlled trial, and it showed promising antitumor activity in terms of pathological responses [60]. NBTXR3 is a first-in-class 50-nm radioenhancer composed of crystalline HfO_2_ NPs that showed the ability to enhance radiation effects in vitro [61]. Preclinical studies have shown that NBTXR3 has a physical mode of action, due to the high atomic number, which does not involve specific biological pathways, thus improving patient outcomes in several types of cancer [10,62]. An in-depth investigation of the mechanisms of radiosensitization using different NPs and comparative assessments of their effectiveness is essential for the development of multiple radiosensitizing agents to target different types of cancers with heterogenous characteristics.

A limitation of this study is that the results are based on single cancer cell line experiments. Doskey et al. [63] demonstrated that the endogenous levels of antioxidant enzymes differ greatly across different tissue types, and the tissues have a wide range of abilities to remove H_2_O_2_. They quantitatively determined such capacities for 10 different normal cell types and 15 different cancer cell lines. They reported that MIA PaCa-2 cells have a low basal capacity to remove H_2_O_2_ and a markedly low catalase activity compared with those of several other cancer cell lines, making them the best model for H_2_O_2_-generating agents. Hence, PAA-TiOxNPs might not show the same efficacy in tumors with a high catalase activity. Further research should be conducted to maximize the efficacy of PAA-TiOxNPs through a combination with catalase inhibitors or by actively targeting tumor tissues by combining with targeted therapy. Next, in the cell-free system, the decrease of O_2_˙ generation within PAA-TiOxNP suspensions under x-irradiation remains unclarified. As far as we searched, data demonstrating the interactions between PAA-TiOxNPs and O_2_˙ is lacking. However, a report by Konaka et al. might be a possible explanation for this phenomenon [64]. They demonstrated that irradiation to TiO_2_ generates O_2_˙. However, O_2_˙ is usually protonated in low pH media and converted to perhydroxyl radical (HO_2_˙) [65]. Immediately, two HO_2_˙ radicals are converted to hydrogen peroxide and singlet oxygen [64,65]. However, further study might be warranted to understand the whole ROS interaction.

## 5. Conclusions

Identification of potentially safe radiosensitizing agents is critical for the development of variable therapeutic approaches that can improve the outcomes of various types of radioresistant tumors. This study compared the different mechanisms of radiosensitization by PAA-TiOxNPs and AuNPs. PAA-TiOxNPs showed the ability to produce H_2_O_2_ molecules in addition to HO˙ radicals in vitro and in vivo. In contrast, AuNPs showed a higher ability to produce HO˙ radicals only. However, the radiosensitizing effect of PAA-TiOxNPs was more effective resulting in more apoptosis and tumor growth inhibition of MIA PaCa-2 human pancreatic cancer xenografts. These findings support the important role of H_2_O_2_ as a mediator of PAA-TiOxNPs’ radiosensitization. Moreover, administration of PAA-TiOxNPs was generally safe and nontoxic and caused no damage to the liver, kidney, lung, or heart tissue.

## Figures and Tables

**Table 1:** Comparison of physicochemical characters of gold nanoparticles (AuNPs) and polyacrylic acid-coated titanium peroxide nanoparticle (PAA-TiOxNPs).

	AuNPs	PAA-TiOxNPs
Shape	Sphere	Sphere
Size with DLS *	50 nm, with a narrow unimodal size distribution	50 nm, with a narrow unimodal size distribution
Surface charge (Zeta potential)	Negative charge	Negative charge (−48.1 ± 14.2 mV)
Surface coating	Surface-bound citrate ions	Polyacrylic acid
References	Nakayama et al. (2020) [30]	Morita et al. (2016) [26] Nakayama et al. (2016) [28] Morita et al. (2018) [29]

* DLS; Dynamic light scattering.

## Supplementary Materials

The following are available online at https://www.mdpi.com/2079-4991/10/6/1125/s1, Figure S1: Concentration of H_2_O_2_ within the nanosolutions. Data are shown as the mean ± standard deviation. Table S1: Raw data for assessment of hydrogen peroxide production in MIA PaCa-2 cells using cH2-DCF fluorescence. Table S2: Raw data for assessment of nanoparticles-induced apoptosis using Tunnel assay. Table S3: Summary of selected studies of AuNPs non-cytotoxicity.

## References

[1] Salata O.V. (2004). Applications of nanoparticles in biology and medicine. J. Nanobiotechnol..

[2] Awasthi R., Roseblade A., Hansbro P.M., Rathbone M.J., Dua K., Bebawy M. (2018). Nanoparticles in cancer treatment: Opportunities and obstacles. Curr. Drug Targets.

[3] Rezvantalab S., Drude N.I., Moraveji M.K., Güvener N., Koons E.K., Shi Y., Lammers T., Kiessling F. (2018). PLGA-based nanoparticles in cancer treatment. Front. Pharmacol..

[4] Kunz-Schughart L.A., Dubrovska A., Peitzsch C., Ewe A., Aigner A., Schellenburg S., Muders M.H., Hampel S., Cirillo G., Iemma F. (2017). Nanoparticles for radiooncology: Mission, vision, challenges. Biomaterials.

[5] Pagáčová E., Štefančíková L., Schmidt-Kaler F., Hildenbrand G., Vičar T., Depeš D., Lee J.H., Bestvater F., Lacombe S., Porcel E. (2019). Challenges and contradictions of metal nano-particle applications for radio-sensitivity enhancement in cancer therapy. Int. J. Mol. Sci..

[6] Fang J., Nakamura H., Maeda H. (2011). The EPR effect: Unique features of tumor blood vessels for drug delivery, factors involved, and limitations and augmentation of the effect. Adv. Drug Deliv. Rev..

[7] Zhang Y., Huang F., Ren C., Liu J., Yang L., Chen S., Chang J., Yang C., Wang W., Zhang C. (2019). Enhanced Radiosensitization by Gold Nanoparticles with Acid-Triggered Aggregation in Cancer Radiotherapy. Adv. Sci..

[8] Liu Z., Tan H., Zhang X., Chen F., Zhou Z., Hu X., Chang S., Liu P., Zhang H. (2018). Enhancement of radiotherapy efficacy by silver nanoparticles in hypoxic glioma cells. Artif. Cells Nanomed. Biotechnol..

[9] Yong Y., Zhang C., Gu Z., Du J., Guo Z., Dong X., Xie J., Zhang G., Liu X., Zhao Y. (2017). Polyoxometalate-based radiosensitization platform for treating hypoxic tumors by attenuating radioresistance and enhancing radiation response. ACS Nano.

[10] Bonvalot S., Le Pechoux C., De Baere T., Kantor G., Buy X., Stoeckle E., Terrier P., Sargos P., Coindre J.M., Lassau N. (2017). First-in-human study testing a new radioenhancer using nanoparticles (NBTXR3) activated by radiation therapy in patients with locally advanced soft tissue sarcomas. Clin. Cancer Res..

[11] Retif P., Pinel S., Toussaint M., Frochot C., Chouikrat R., Bastogne T., Barberi-Heyob M. (2015). Nanoparticles for radiation therapy enhancement: The key parameters. Theranostics.

[12] Hainfeld J.F., Slatkin D.N., Smilowitz H.M. (2004). The use of gold nanoparticles to enhance radiotherapy in mice. Phys. Med. Biol..

[13] Borran A.A., Aghanejad A., Farajollahi A., Barar J., Omidi Y. (2018). Gold nanoparticles for radiosensitizing and imaging of cancer cells. Radiat. Phys. Chem..

[14] Haume K., Rosa S., Grellet S., Śmiałek M.A., Butterworth K.T., Solov’yov A.V., Prise K.M., Golding J., Mason N.J. (2016). Gold nanoparticles for cancer radiotherapy: A review. Cancer Nanotechnol..

[15] Tang Y., Shen Y., Huang L., Lv G., Lei C., Fan X., Lin F., Zhang Y., Wu L., Yang Y. (2015). In vitro cytotoxicity of gold nanorods in A549 cells. Environ. Toxicol. Pharmacol..

[16] Rosa S., Connolly C., Schettino G., Butterworth K.T., Prise K.M. (2017). Biological mechanisms of gold nanoparticle radiosensitization. Cancer Nano.

[17] Saberi A., Shahbazi-Gahrouei D., Abbasian M., Fesharaki M., Baharlouei A., Arab-Bafrani Z. (2017). Gold nanoparticles in combination with megavoltage radiation energy increased radiosensitization and apoptosis in colon cancer HT-29 cells. Int. J. Radiat. Biol..

[18] Li T., Yang C., Zhang G., Huang J., Lyu J., Qin S. (2018). Radiosensitization and micro CT imaging of multifunctional gold nanoparticles in lung adenocarcinoma A549 cell: An in vivo animal study. Int. J. Radiat. Oncol. Biol. Phys..

[19] Chithrani B.D., Ghazani A.A., Chan W.C. (2006). Determining the size and shape dependence of gold nanoparticle uptake into mammalian cells. Nano Lett..

[20] Lee E., Jeon H., Lee M., Ryu J., Kang C., Kim S., Jung J., Kwon Y. (2019). Molecular origin of AuNPs-induced cytotoxicity and mechanistic study. Sci. Rep..

[21] Adewale O.B., Davids H., Cairncross L., Roux S. (2019). Toxicological behavior of gold nanoparticles on various models: Influence of physicochemical properties and other factors. Int. J. Toxicol..

[22] Doudi M., Setorki M. (2014). The acute liver injury in rat caused by gold nanoparticles. Nanomed. J..

[23] Abdelhalim M.A.K., Qaid H.A., Al-Mohy Y., Al-Ayed M.S. (2018). Effects of quercetin and arginine on the nephrotoxicity and lipid peroxidation induced by gold nanoparticles in vivo. Int. J. Nanomed..

[24] Abdelhalim M.A.K., Moussa S.A.A., Qaid H.A., Al-Ayed M.S. (2018). Potential effects of different natural antioxidants on inflammatory damage and oxidative-mediated hepatotoxicity induced by gold nanoparticles. Int. J. Nanomed..

[25] Abdelhalim M.A.K., Qaid H.A., Al-Mohy Y.H., Ghannam M.M. (2020). The Protective Roles of Vitamin E and alpha-Lipoic Acid Against Nephrotoxicity, Lipid Peroxidation, and Inflammatory Damage Induced by Gold Nanoparticles. Int. J. Nanomed..

[26] Morita K., Miyazaki S., Numako C., Ikeno S., Sasaki R., Nishimura Y., Ogino C., Kondo A. (2016). Characterization of titanium dioxide nanoparticles modified with polyacrylic acid and H_2_O_2_ for use as a novel radiosensitizer. Free Radic. Res..

[27] Kanehira K., Banzai T., Ogino C., Shimizu N., Kubota Y., Sonezaki S. (2008). Properties of TiO2-polyacrylic acid dispersions with potential for molecular recognition. Colloids Surf. B Biointerfaces.

[28] Nakayama M., Sasaki R., Ogino C., Tanaka T., Morita K., Umetsu M., Ohara S., Tan Z., Nishimura Y., Akasaka H. (2016). Titanium peroxide nanoparticles enhanced cytotoxic effects of X-ray irradiation against pancreatic cancer model through reactive oxygen species generation in vitro and in vivo. Radiat. Oncol..

[29] Morita K., Suzuki T., Nishimura Y., Matsumoto K., Numako C., Sato K., Nakayama M., Sasaki R., Ogino C., Kondo A. (2018). In vivo tissue distribution and safety of polyacrylic acid-modified titanium peroxide nanoparticles as novel radiosensitizers. J. Biosci. Bioeng..

[30] Nakayama M., Akasaka H., Geso M., Morita K., Yada R., Uehara K., Sasaki R. (2020). Utilisation of the chemiluminescence method to measure the radiation dose enhancement caused by gold nanoparticles: A phantom-based study. Radiat. Meas..

[31] Shimizu Y., Mukumoto N., Idrus N., Akasaka H., Inubushi S., Yoshida K., Miyawaki D., Ishihara T., Okamoto Y., Yasuda T. (2019). Amelioration of radiation enteropathy by dietary supplementation with reduced coenzyme Q10. Adv. Radiat. Oncol..

[32] Setsukinai K., Urano Y., Kakinuma K., Majima H.J., Nagano T. (2003). Development of novel fluorescence probes that can reliably detect reactive oxygen species and distinguish specific species. J. Biol. Chem..

[33] Akasaka H., Mizushina Y., Yoshida K., Ejima Y., Mukumoto N., Wang T., Inubushi S., Nakayama M., Wakahara Y., Sasaki R. (2016). MGDG extracted from spinach enhances the cytotoxicity of radiation in pancreatic cancer cells. Radiat. Oncol..

[34] Sasaki R., Shirakawa T., Zhang Z.J., Tamekane A., Matsumoto A., Sugimura K., Matsuo M., Kamidono S., Gotoh A. (2001). Additional gene therapy with Ad5CMV-p53 enhanced the efficacy of radiotherapy in human prostate cancer cells. Int. J. Radiat. Oncol. Biol. Phys..

[35] Imlay J.A., Chin S.M., Linn S. (1988). Toxic DNA damage by hydrogen peroxide through the Fenton reaction in vivo and in vitro. Science.

[36] Yamaguchi H., Uchihori Y., Yasuda N., Takada M., Kitamura H. (2005). Estimation of yields of OH radicals in water irradiated by ionizing radiation. J. Radiat. Res..

[37] Ogawa Y. (2016). Paradigm shift in radiation biology/radiation oncology-exploitation of the “H2O2 effect” for radiotherapy using low-LET (linear energy transfer) radiation such as X-rays and high-energy electrons. Cancers.

[38] Ogawa Y., Ue H., Tsuzuki K., Tadokoro M., Miyatake K., Sasaki T., Yokota N., Hamada N., Kariya S., Hitomi J. (2008). New radiosensitization treatment (KORTUC I) using hydrogen peroxide solution-soaked gauze bolus for unresectable and superficially exposed neoplasms. Oncol. Rep..

[39] Ogawa Y., Kubota K., Ue H., Kataoka Y., Tadokoro M., Miyatake K., Suzuki K., Yamanishi T., Itoh S., Hitomi J. (2009). Phase I study of a new radiosensitizer containing hydrogen peroxide and sodium hyaluronate for topical tumor injection: A new enzyme-targeting radiosensitization treatment, Kochi oxydol-radiation therapy for unresectable carcinomas, Type II (KORTUC II). Int. J. Oncol..

[40] Coulter J., Jain S., Butterworth K.T., Taggart L.E., Dickson G.R., McMahon S.J., Hyland W.B., Muir M.F., Trainor C., Hounsell A.R. (2012). Cell type-dependent uptake, localization, and cytotoxicity of 1.9 nm gold nanoparticles. Int. J. Nanomed..

[41] Cui L., Her S., Borst G.R., Bristow R.G., Jaffray D.A., Allen C. (2017). Radiosensitization by gold nanoparticles: Will they ever make it to the clinic?. Radioth. Oncol..

[42] Pan Y., Leifert A., Ruau D., Neuss S., Bornemann J., Schmid G., Brandau W., Simon U., Jahnen-Dechent W. (2009). Gold nanoparticles of diameter 1.4 nm trigger necrosis by oxidative stress and mitochondrial damage. Small.

[43] Jia Y.-P., Ma B.-Y., Wei X.-W., Qian Z.-Y. (2017). The in vitro and in vivo toxicity of gold nanoparticles. Chin. Chem. Lett..

[44] Goodman C.M., McCusker C.D., Yilmaz T., Rotello V.M. (2004). Toxicity of gold nanoparticles functionalized with cationic and anionic side chains. Bioconjug. Chem..

[45] Connor E., Mwamuka J., Gole A., Murphy C., Wyatt M. (2005). Gold nanoparticles are taken up by human cells but do not cause acute cytotoxicity. Small.

[46] Takahashi H., Niidome Y., Niidome T., Kenji Kaneko K., Kawasaki H., Yamada S. (2006). Modification of gold nanorods using phosphatidylcholine to reduce cytotoxicity. Langmuir.

[47] Niidome T., Yamagata M., Okamoto Y., Akiyama Y., Takahashi H., Kawano T., Katayama Y., Niidome Y. (2006). PEG-modified gold nanorods with a stealth character for in vivo applications. J. Control. Release.

[48] Hauck T., Ghazani A., Chan W. (2008). Assessing the effect of surface chemistry on gold nanorod uptake, toxicity, and gene expression in mammalian cells. Small.

[49] Sonavane G., Tomoda K., Makino K. (2008). Biodistribution of colloidal gold nanoparticles after intravenous administration: Effect of particle size. Colloids Surf. B.

[50] Conde J., Larguinho M., Cordeiro A., Raposo L.R., Costa P.M., Santos S., Diniz M.S., Fernandes A.R., Pedro V., Baptista P.V. (2014). Gold-nanobeacons for gene therapy: Evaluation of genotoxicity, cell toxicity and proteome profiling analysis. Nanotoxicology.

[51] Rambanapasi C., Zeevaart R.J., Buntting H., Bester C., Kotze D., Hayeshi R., Grobler A. (2016). Bioaccumulation and subchronic toxicity of 14 nm gold nanoparticles in rats. Molecules.

[52] Mukherjee S., Sau S., Madhuri D., Bollu V.S., Madhusudana K., Sreedhar B., Banerjee R., Patra C.R. (2016). Green synthesis and characterization of monodispersed gold nanoparticles: Toxicity study, delivery of doxorubicin and its bio-distribution in mouse model. J. Biomed. Nanotechnol..

[53] Ogawa Y., Takahashi T., Kobayashi T., Kariya S., Nishioka A., Ohnishi T., Saibara T., Hamasato S., Tani T., Seguchi H. (2003). Apoptotic-resistance of the human osteosarcoma cell line HS-Os-1 to irradiation is converted to apoptotic-susceptibility by hydrogen peroxide: A potent role of hydrogen peroxide as a new radiosensitizer. Int. J. Mol. Med..

[54] Kariya S., Sawada K., Kobayashi T., Karashima T., Shuin T., Nishioka A., Ogawa Y. (2009). Combination treatment of hydrogen peroxide and X-rays induces apoptosis in human prostate cancer PC-3 cells. Int. J. Radiat. Oncol. Biol. Phys..

[55] Fang Y., Moore B.J., Bai Q., Cook K.M., Herrick E.J., Nicholl M.B. (2013). Hydrogen peroxide enhances radiation-induced apoptosis and inhibition of melanoma cell proliferation. Anticancer Res..

[56] Kerher J.P. (2000). The Haber–Weiss reaction and mechanisms of toxicity. Toxicology.

[57] Ju H.Q., Gocho T., Aguilar M., Wu M., Zhuang Z.N., Fu J., Yanaga K., Huang P., Chiao P.J. (2015). Mechanisms of overcoming intrinsic resistance to gemcitabine in pancreatic ductal adenocarcinoma through the redox modulation. Mol. Cancer Ther..

[58] Ju H.Q., Lu Y.X., Chen D.L., Tian T., Mo H.Y., Wei X.L., Liao J.W., Wang F., Zeng Z.L., Pelicano H. (2016). Redox regulation of stem-like cells though the CD44v-xCT axis in colorectal cancer: Mechanisms and therapeutic implications. Theranostics.

[59] Trachootham D., Alexandre J., Huang P. (2009). Targeting cancer cells by ROS mediated mechanisms: A radical therapeutic approach?. Nat. Rev. Drug Discov..

[60] Bonvalot S., Rutkowski P.L., Thariat J., Carrère S., Ducassou A., Sunyach M.P., Agoston P., Hong A., Mervoyer A., Rastrelli M. (2019). NBTXR3, a first-in-class radioenhancer hafnium oxide nanoparticle, plus radiotherapy versus radiotherapy alone in patients with locally advanced soft-tissue sarcoma (Act.In.Sarc): A multicentre, phase 2-3, randomised, controlled trial. Lancet Oncol..

[61] Marill J., Anesary N.M., Zhang P., Vivet S., Borghi E., Levy L., Pottier A. (2014). Hafnium oxide nanoparticles: Toward an in vitro predictive biological effect?. Radiat. Oncol..

[62] Maggiorella L., Barouch G., Devaux C., Pottier A., Deutsch E., Bourhis J., Borghi E., Levy L. (2012). Nanoscale radiotherapy with hafnium oxide nanoparticles. Future Oncol..

[63] Doskey C.M., Buranasudja V., Wagner B.A., Wilkes J.G., Du J., Cullen J.J., Buettner G.R. (2016). Tumor cells have decreased ability to metabolize H2O2: Implications for pharmacological ascorbate in cancer therapy. Redox Biol..

[64] Konaka R., Kasahara E., Dunlap W.C., Yamamoto Y., Chien K.C., Inoue M. (1999). Irradiation of Titanium Dioxide Generates Both Singlet Oxygen and Superoxide Anion. Free Radic. Biol. Med..

[65] Collin F. (2019). Chemical Basis of Reactive Oxygen Species Reactivity and Involvement in Neurodegenerative Diseases. Int. J. Mol. Sci..

