# Supplementary material for: A Comparative Assessment of Mechanisms and Effectiveness of Radiosensitization by Titanium Peroxide and Gold Nanoparticles
Source: Nanomaterials (Basel). 2020 Jun 7;10(6):1125. doi: 10.3390/nano10061125 (PMC7353194; doi:10.3390/nano10061125)
Supplement: Supplementary file 1 [file nanomaterials-10-01125-s001.zip › supplementary tables.docx]

Table S1. Raw data for assessment of hydrogen peroxide production in MIA PaCa-2 cells using cH2-DCF fluorescence.

|  | No of DCF positive cells/ total No. of cells | | | | | | Mean |
| --- | --- | --- | --- | --- | --- | --- | --- |
|  | Sample 1 | | Sample 2 | | Sample 3 | | %DCF positive cells |
| Control | 18/373 | 4.8 (%) | 15/293 | 5.1 (%) | 6/204 | 2.9 (%) | 4.3 (%) |
| AuNPs | 27/344 | 7.8 (%) | 17/108 | 15.7 (%) | 29/189 | 15.3 (%) | 13.0 (%) |
| TiOxNPs | 46/155 | 29.7 (%) | 31/96 | 32.3 (%) | 52/172 | 30.2 (%) | 30.7 (%) |
| 5 Gy | 56/236 | 23.7 (%) | 30/176 | 17.0 (%) | 25/231 | 10.8 (%) | 17.2 (%) |
| AuNPs+5Gy | 38/144 | 26.4 (%) | 54/190 | 28.4 (%) | 45/190 | 23.7 (%) | 26. 2 (%) |
| TiOxNPs+5Gy | 155/250 | 62 (%) | 105/184 | 57.1 (%) | 144/250 | 57.6 (%) | 58.9 (%) |

Table S2. Raw data for assessment of nanoparticles-induced apoptosis using Tunnel assay

|  | No of apoptotic cells/ total No. of cells | | | | | | Mean |
| --- | --- | --- | --- | --- | --- | --- | --- |
|  | Sample 1 | | Sample 2 | | Sample 3 | | **%** apoptotic cells |
| Control | 5/146 | 3.4 (%) | 8/156 | 5.1 (%) | 3/74 | 4.1 (%) | 4.2 (%) |
| AuNPs | 19/221 | 8.6 (%) | 15/266 | 5.6 (%) | 15/198 | 7.6 (%) | 7.3 (%) |
| TiOxNPs | 25/196 | 12.8 (%) | 20/172 | 11.6 (%) | 16/143 | 11.2 (%) | 11.9 (%) |
| 5 Gy | 15/90 | 16.7 (%) | 25/183 | 13.7 (%) | 42/341 | 12.3 (%) | 14.2 (%) |
| AuNPs+5Gy | 27/182 | 14.8 (%) | 30/213 | 14.1 (%) | 35/211 | 16.6 (%) | 15.2 (%) |
| TiOxNPs+5Gy | 51/231 | 22.1 (%) | 51/215 | 23.7 (%) | 49/251 | 19.5 (%) | 21.8 (%) |

Table S3. Summary of selected studies of AuNPs non-cytotoxicity.

| AuNPs | Size (nm) | Surface coating/functionalization | Shape | Cell lines/　animals | Methods of evaluation | Findings | Reference |
| --- | --- | --- | --- | --- | --- | --- | --- |
| Cationic and anionic mixed monolayer protected Au clusters (MMPCs) | ~6 | Ammonium functionalized nanoparticle (MMPC 1) and carboxylate-substituted nanoparticle (MMPC 2) |  | In vitro, Cos-1 and red blood cells | MTT viability assay | cationic particles are moderately toxic, whereas anionic particles are quite non-toxic due to cell membrane lysis by the nanoparticles | Goodman et.al., 2004. [44] |
| Surface modified AuNPs | ~4, 12, and 18 | Cysteine and citrate-capped 4-nm nanoparticles, glucose-reduced 12-nm nanoparticles and citrate, biotin, and cetyltrimethylammonium bromide | Nanospheres | In vitro, Human K562 leukemia cell line | MTT viability assay | AuNPs with surface modifiers are not toxic to human cells, despite being taken up into the cells. However, gold-salt (AuCl4) precursor showed some toxicity | Connor et. al., 2005. [45] |
| Au nanorods (PC-NRs) | Average length and width were 65 nm and 11 nm. | Hexadecyltrimethylammonium bromide replaced with phosphatidylcholine | Nanorods | In vitro, Hela cells | MTT viability assay | cytotoxicity of the PC-NRs was negligible | Takahashi et. al., 2006. [46] |
| PEG-modified Au nanorods | Average length and width were 65 nm and 11 nm. | CTAB removed and modified with polyethyleneglycol | Nanorods | In vitro, Hela cells. In vivo, intravenous injection of mice Male ddY mice examined for NP biodistribution | MTT viability assay. In vivo, quantitative analysis of AuNPs in blood and tissues was performed by ICP mass spectrometry | PEG-modified Au nanorods showed low cytotoxicity in vitro and there was no accumulation in major organs except for the liver | Niidome et. al., 2006. [47] |
| PDADMAC-coated gold nanorods |  | Coating with poly(diallyldimethylammonium chloride)-poly(4-styrenesulfonic acid) system | Nanorods | In vitro, Hela cells | cell viability assay and gene expression analysis | AuNPs showed low toxicity and there were no major changes in gene expression between the control and PDADMAC-treated cells | Hauck et. al., 2008. [48] |
| AuNPs | 15, 50, 100, and 200 | Citrate surface coating | Nanospheres | In vivo, intravenous injection of male ddY mice to assess NP biodistribution | NP concentration of different sizes were measured in blood and tissues including heart, liver, lung, spleen, kidney, stomach, pancreas, and brain | Tissue distribution of AuNPs is size-dependent. Large amounts of 15 nm AuNPs were detected in all tissues including the brain; however, small amount of 200 nm AuNPs were detected in blood, brain, stomach, and pancreas | Sonavane et. al., 2008. [49] |
| antisense Au-nanobeacon | 14.6 ± 1.7 nm by TEM.  16.7 ± 5.9 nm by DLS. | Functionalized with a PEG and contain a stem-looped oligonucleotide of unrelated sequence double-labeled with 3′-Cy3 and 5′-Thiol-C6 | Nanosphere | In vitro, HCT-116 cells | MTT assay, genotoxicity assay (Comet assay and frequency of nuclear abnormalities) and oxidative stress assay (glutathione -S- transferase measurement) Proteome profiling | The proposed nanotheranostics strategy does not exhibit significant toxicity | Conde et. al., 2013. [50] |
| AuNPs | 14 ± 1.2 nm | Citrate surface coating | Nanospheres | In vivo, intravenous injection of male Sprague Dawley rats (liver, lungs, skeleton, spleen, and carcass) | Biodistribution of AuNPs was determined using neutron activation analysis, blood markers for kidney and liver damage and histopathological assessment | There was no observable acute or subchronic toxicity despite the accumulation of AuNPs in organs/tissues | Rambanapasi et. al, 2016. [51] |
| b-Au-PP-400 | 5–15 nm by TEM; 55.4 nm by DLs. | Coated with various biomolecules and proteins originating from the Peltophorum pterocarpum extract | Nanospheres | In vitro, normal endothelial cells (HUVEC, ECV 304). In vivo, intraperitoneal (IP) injections in C57BL6/J mice | In vitro, MTT viability assay. In vivo, biochemical blood tests and histological evaluation of liver, lung, kidney, spleen, brain, heart, thymus, and uterus | In vitro, no inhibition of cell proliferation was observed.  In vivo, no major significant biochemical or histopathological toxicities were observed | Mukherjee et. al., 2016. [52] |
| Modified AuNPs | Range from 24.7 to 49.1 nm. | Citrate-coated and modified with various thiol ligands by place-exchange reaction | Nanospheres | In vitro, HeLa and Normal human dermal fibroblasts | MTT, trypan blue, colony forming and DNA damage assays | cationic AuNPs exhibit a range of cytotoxicity from being non-toxic to being severely toxic while neutral and anionic AuNPs were not noticeably cytotoxic | Lee et. al., 2019. [20] |
