# Supplementary figures and images for: A Comparative Assessment of Mechanisms and Effectiveness of Radiosensitization by Titanium Peroxide and Gold Nanoparticles
Source: Nanomaterials (Basel). 2020 Jun 7;10(6):1125. doi: 10.3390/nano10061125 (PMC7353194; doi:10.3390/nano10061125)

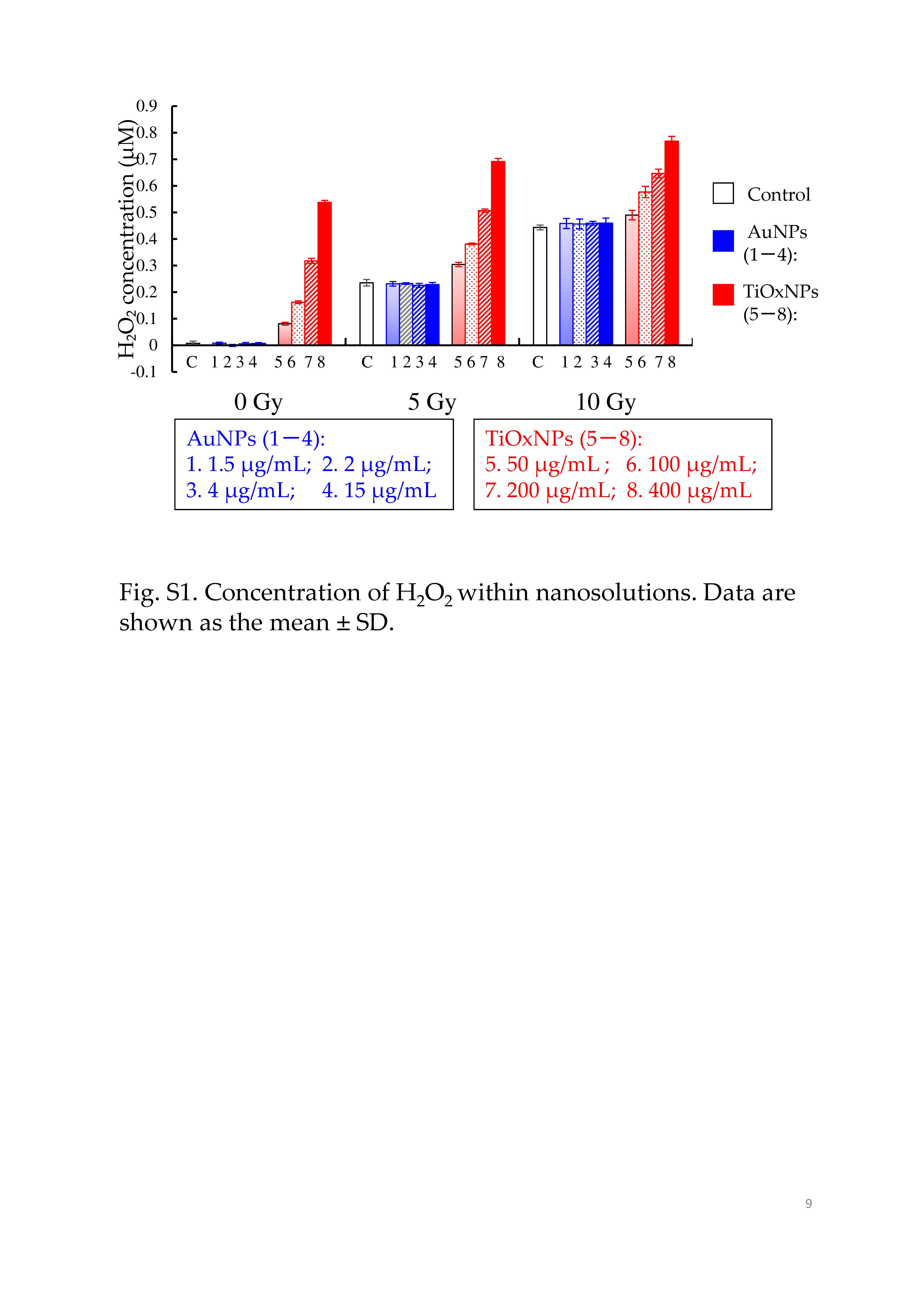

Supplement: Supplementary file 1 [file nanomaterials-10-01125-s001.zip › figure S1.jpg]
